# Crosstalk between neuroinflammation and oxidative stress in epilepsy

**DOI:** 10.3389/fcell.2022.976953

**Published:** 2022-08-10

**Authors:** Timothy Fabisiak, Manisha Patel

**Affiliations:** Department of Pharmaceutical Sciences, Skaggs School of Pharmacy and Pharmaceutical Sciences, University of Colorado Anschutz Medical Center, Aurora, CO, United States

**Keywords:** neuroinflammation, oxidative stress, redox, epilepsy, mitochondria, NADPH oxidase

## Abstract

The roles of both neuroinflammation and oxidative stress in the pathophysiology of epilepsy have begun to receive considerable attention in recent years. However, these concepts are predominantly studied as separate entities despite the evidence that neuroinflammatory and redox-based signaling cascades have significant crosstalk. Oxidative post-translational modifications have been demonstrated to directly influence the function of key neuroinflammatory mediators. Neuroinflammation can further be controlled on the transcriptional level as the transcriptional regulators NF-KB and nrf2 are activated by reactive oxygen species. Further, neuroinflammation can induce the increased expression and activity of NADPH oxidase, leading to a highly oxidative environment. These factors additionally influence mitochondria function and the metabolic status of neurons and glia, which are already metabolically stressed in epilepsy. Given the implication of this relationship to disease pathology, this review explores the numerous mechanisms by which neuroinflammation and oxidative stress influence one another in the context of epilepsy. We further examine the efficacy of treatments targeting oxidative stress and redox regulation in animal and human epilepsies in the literature that warrant further investigation. Treatment approaches aimed at rectifying oxidative stress and aberrant redox signaling may enable control of neuroinflammation and improve patient outcomes.

## Introduction

There are 31 types of epilepsy syndromes classified by the International League Against Epilepsy (Epilepsy Syndromes, 2022) based on numerous features such as seizure type, epilepsy presentation, and etiology (Scheffer et al., 2017). This disease complexity presents a challenge in the treatment of epilepsy, where available anti-seizure medications (ASMs) that are tailored to the epilepsy syndrome generally only achieve a 30–50% responder rate (Löscher and Klein, 2021). The prevalence of drug-resistant epilepsy highlights a need to further understand underlying mechanisms in epilepsy such as neuroinflammation and consequential signaling cascades, as reviewed further elsewhere (Vezzani et al., 2019). Yet more recently, oxidative stress and redox dysregulation have also emerged as hallmarks in the epilepsy literature (Patel, 2004; Pearson-Smith & Patel, 2017; Geronzi et al., 2018).

**SCHEME 1:**
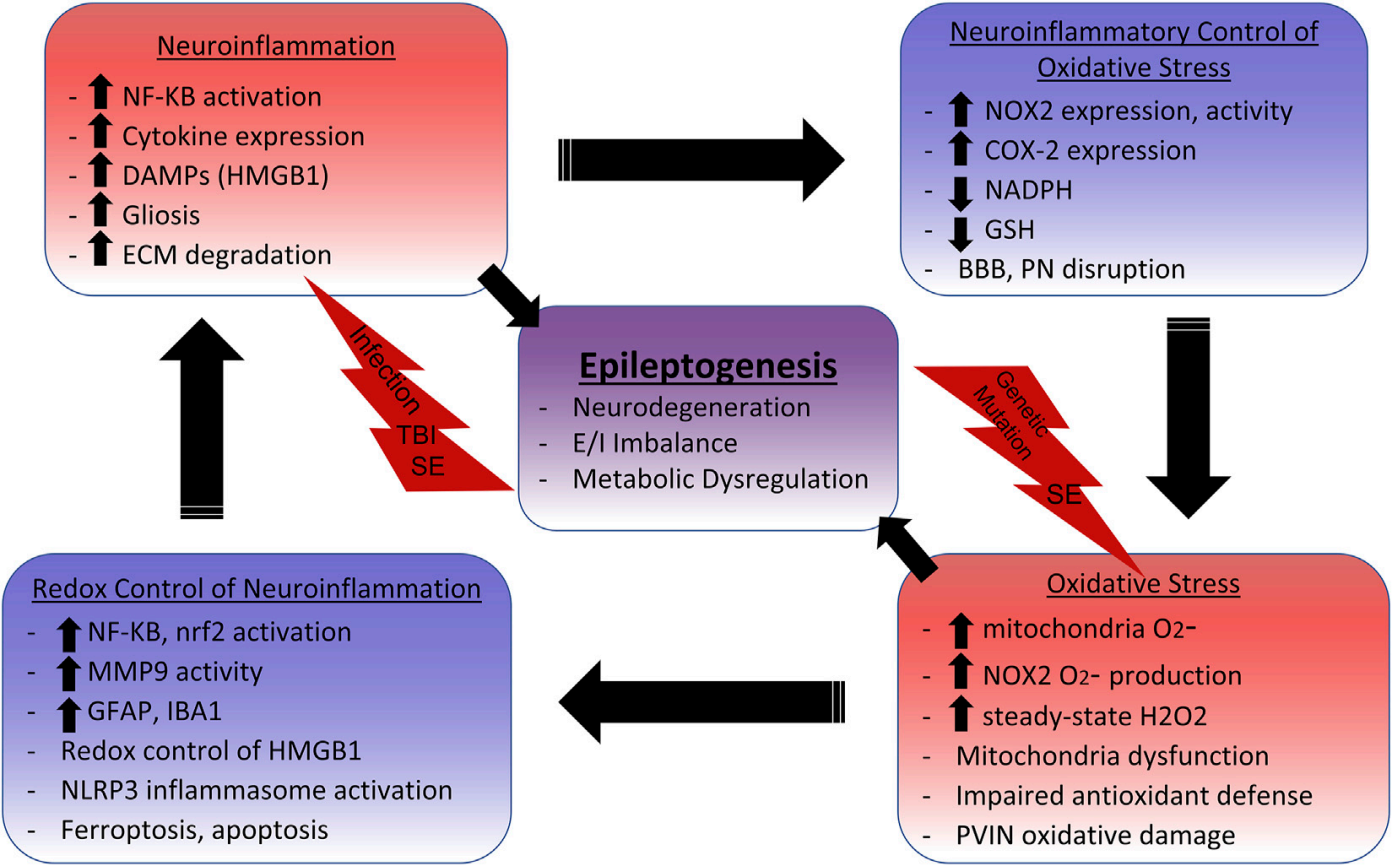
Neuroinflammation and Oxidative Stress Cycle in Epilepsy.Initial insults such as systemic infection, traumatic brain injury (TBI), status epilepticus (SE), and genetic mutations initiate inflammatory and oxidative signaling cascades. NF-KB activation leads to the production of proinflammatory cytokines such as IL-1β, TNFα, and IL-6, which have been shown to i nduce gliosis, mitochondrial dysfunction, and glutathione (GSH) depletion. GSH depletion impairs cellular and mitochondrial antioxidant defenses, enabling aberrant oxidative signaling and damage. NAPDH Oxidase 2 (NOX2) upregulation also commonly occurs with neuroinflammation. NOX2 utilizes NADPH, which is needed for the reductive abilities of antioxidant systems like thioredoxin reductase and glutathione peroxidase, to produce superoxide (O_2_^-^)_._ O_2_^-^is primarily enzymatically converted to the redox-signaling reactive H_2_O_2_. The increased levels of steady-state H_2_O_2_ leads to the activation of numerous redox-sensitive pathways that influence neuroinflammation. For example, NF-KB activation and subsequent microglia activation relies on NOX2 activity, indicating that reactive oxygen species (ROS) production perpetuates neuroinflammation. Nrf2 is also activated by ROS to increase expression of the antioxidant response element (ARE) to combat oxidative damage and neuroinflammation, making it an attractive therapeutic target. The danger-associated molecular pattern (DAMP) HMGB1 is released in response to neuronal damage, which signals through TLR4 to activate NF-KB, induce gliosis, and increase NOX2 and COX-2 expression. Reduced HMGB1 has chemoattractant properties, but adjacent cysteine residues act as a redox switch where oxidation to a disulfide form increases affinity for TLR4 and induces cytokine like properties. The NLRP3 inflammasome, which activates IL-1β, can also by activated by ROS from the mitochondria and NOX. Extracellular matrix (ECM) digesting proteinases such as matrix metalloproteinases (MMPs) are also upregulated in the neuroinflammatory environment. This can lead to the disruption of the blood brain barrier (BBB) and perineuronal nets (PNs) surrounding inhibitory parvalbumin interneurons (PVINs). MMP9 has demonstrated redox-sensitive activation, which is associated with the loss of PNs and death of PVINs. Mitochondria dysfunction can result from excessive mitochondrial ROS (mtROS) which not only accounts for metabolic alterations, but can lead to inflammation-inducing events such as astrogliosis, apoptosis, and lipid peroxidase mediated death (ferroptosis). Metabolic dysfunction, excitatory/inhibitory (E/I) imbalance, and neurodegeneration can all result from these oxidative and inflammatory processes which ultimately contribute to epileptogenesis.

Interestingly, in human epilepsies and animal epilepsy models, various biomarkers of both oxidative stress and neuroinflammation are elevated in the brain and periphery. Neuroinflammation and reactive oxygen species (ROS) production are both prominent effectors of signaling and are therefore heavily regulated. It is not unexpected for these prominent cell signaling processes to have influence on the regulation of one another. As an evolutionarily conserved defense mechanism, inflammation in response to infection or injury promotes and utilizes ROS to kill pathogens or enact rapid, local signaling. At physiological levels, ROS is an important second messenger that modulates neuroinflammation at numerous stages through redox-sensitive mechanisms. Further, excessive ROS production or redox dysregulation under oxidative stress damages cells and produces danger signals that incite neuroinflammation. As oxidative stress and neuroinflammatory pathways have considerable crosstalk (See Scheme 1), there is an unmet need for further research exploring this relationship in the pathophysiology of epilepsy. This can enable the development of novel redox-based therapeutics, beyond traditional ASMs which primarily aim to rectify neural excitatory/inhibitor imbalances at the synapse, to control neuroinflammation and in turn, epilepsy and/or its comorbidities.

## Oxidative stress in epilepsy

The brain is particularly susceptible to oxidative damage due to its high metabolic demand and per weight rate of oxygen consumption. It has long been recognized that ictal cerebral hypermetabolism is characteristic of seizure disorders, where increased cerebral oxygen (Meyer et al., 1966) and glucose consumption (Ingvar and Siesjö, 1983) are observed during seizure activity in experimental epilepsy models and in humans (During et al., 1994). These metabolic stresses lead to interictal metabolic dysfunction which is evidenced by glucose hypometabolism in chronic epilepsy (Guo et al., 2009) and mitochondrial metabolic deficits resulting from inhibition of mitochondrial enzyme complexes in animal and human epilepsies (Kunz et al., 2000, 1999). It has been demonstrated that mitochondrial reactive oxygen species (mtROS) damage mitochondrial electron transport chain complex I, subsequently decreasing mitochondrial oxidative phosphorylation (Ryan et al., 2012; Rowley et al., 2015). Further, pharmacological inhibition of mitochondrial enzyme complexes results in decreased oxidative phosphorylation and seizure activity (Fleck et al., 2004).

There are numerous indications that increased ROS production occurs across epilepsy models, as evidenced by oxidative damage and impaired redox status. In addition to Complex I inhibition in humans and animal models of temporal lobe epilepsy (TLE), the redox regulated TCA cycle enzymes aconitase and *α*-ketoglutarate dehydrogenase have been shown to be inactivated following status epilepticus (SE) in rats (Cock et al., 2002). Increased mitochondria oxidative phosphorylation is also shown to increase complex III mediated mtROS production (Malinska et al., 2010). Mitochondrial DNA (mtDNA) oxidative damage has also been found in the kainate model of TLE, along with increases in mitochondrial H_2_O_2_ (Jarrett et al., 2008a). Further, numerous other animal models and human epilepsies revealed depleted glutathione (GSH) levels, paired with increased oxidized glutathione (glutathione disulfide or GSSG) in the hippocampus or neocortex, indicative of an oxidative environment (Rumià et al., 2013; Cárdenas-Rodríguez et al., 2014; Pearson-Smith and Patel, 2017). As GSH acts as a major antioxidant in the brain, this impaired GSH redox status can lead to further oxidative damage and redox dysregulation, which is associated with cognitive deficits, neuronal death, and mortality in animal epilepsy models. Indeed, loss of glutathione peroxidase 4, a oxidation-resistant selenoprotein that utilizes GSH, leads to lipid peroxidation and subsequent ferroptosis, which is also found in epilepsy models (Ingold et al., 2018; Cai and Yang, 2021).

The NADPH oxidase (NOX) family of enzymes is another relevant major source of ROS in the context of epilepsy as evidenced by activation of NOX2 by kainate-induced SE (Patel et al., 2005). NOX enzymes utilize NADPH to produce superoxide (O_2_^−^), which then undergoes primarily enzymatic dismutation to H_2_O_2._ Interestingly, it has been demonstrated that NMDA receptor activation, which is increased due to excessive extracellular glutamate in epilepsy, increases O_2_^−^ and subsequent H_2_O_2_ production via NOX induction (Brennan et al., 2009; Kovac et al., 2014). Further, NOX activation has been shown to be a sufficient trigger of seizure activity, while NOX inhibition reduced hyperactivity in multiple animal models of epilepsy (Malkov et al., 2019). It is argued that NOX2, the primary isoform in microglia, is thought to be the major contributor of SE-induced ROS production in acquired epilepsies, which has also been linked to glucose hypometabolism in epileptogenesis (Zilberter et al., 2022).

Biomarkers of oxidative stress in human epilepsies have recently received further attention. In the blood of patients with SE, lower levels of SOD, catalase, GSH, and total antioxidant capacity were found (Kalita et al., 2019). In drug-resistant complex partial seizure patient blood, there was an inverse correlation of vitamin C and positive correlation of the oxidative damage marker 3-nitrotyrosine to seizure frequency (Lorigados Pedre et al., 2018). Justifiably, therapies targeting oxidative stress are currently under investigation. In rat electrical status epilepticus, GSH increasing drug treatment was neuroprotective and reduced seizure frequency (Pauletti et al., 2019). Inhibition of KEAP1 by RTA 408 disinhibits the antioxidant transcription factor nrf2 and has neuroprotective and seizure reducing effects in the kainic model of TLE (Shekh-Ahmad et al., 2018). Treatment with a NOX inhibitor, a catalytic antioxidant, and a scavenger of reactive oxidized compounds gamma-ketoaldehydes, have all been shown to prevent experimental TLE seizure-induced neuronal death (Kim et al., 2013; Pearson et al., 2017; Pearson-Smith et al., 2017). In humans, Vitamin E in conjunction with ASMs has been shown to reduce seizure frequency and oxidative stress (Mehvari et al., 2016). Further, the high-fat low-carbohydrate ketogenic diet (KD), which shifts metabolism towards fatty acid oxidation and away from glycolysis, also has antioxidant properties which may contribute to its clinical efficacy in Dravet Syndrome (Caraballo et al., 2005). As a treatment for experimental or genetic epilepsies in rodents, this diet can reduce ROS production, activate nrf2, and increase the synthesis of GSH (Jarrett et al., 2008b; Milder and Patel, 2012).

Clearly, oxidative stress is associated with epilepsy which may be a consequence of the disease. Interestingly, there is further evidence indicating that mitochondrial dysfunction and oxidative stress contribute to epilepsy pathogenesis (Patel, 2004). The most robust links between oxidative stress or mitochondrial dysfunction and epilepsy are clear in mitochondrial encephalopathies such as myclonic epilepsy with ragged-red fibers (MERFF) and Leigh syndrome. These disorders are characterized by mtDNA mutations that impair mitochondria electron transport chain enzyme complexes, which can result in increased ROS production, oxidative damage, and decreased ATP production (Quintana et al., 2010; Wu et al., 2010; Wojtala et al., 2017). Other genetic epilepsies such as Dravet Syndrome are further associated with mitochondrial dysfunction (Kumar et al., 2016; Banerji et al., 2021). Genetic knockouts in mice of mitochondrial superoxide dismutase (SOD2), which detoxifies O_2_^−^ to H_2_O_2_, increases susceptibility to spontaneous and induced seizures (Liang and Patel, 2004; Liang et al., 2012). Further, the forebrain neuron-specific conditional knockout of SOD2 also leads to the presentation of epilepsy in mice (Rowley et al., 2015). Together, this body of work demonstrates that oxidative stress is not only a consequence of epilepsy, but also a cause.

## Concomitance of neuroinflammation and oxidative stress in epilepsy

Seizure inciting events such as infection or traumatic brain injury (TBI) that initiate proinflammatory cascades are also linked to oxidative stress. Post-traumatic epilepsy (PTE) develops in as many as 50% of TBI patients, and TBI and chronic epilepsy brain tissue both display elevated markers of oxidative stress in addition to cytokines and neurodegeneration (Vezzani et al., 2008; Helmy et al., 2011; Terrone et al., 2019). NOX2 expression is additionally elevated in the brain following TBI and in epilepsy as reviewed by Ma et al., 2018. In experimental models of acquired epilepsy, such as chemically induced status epilepticus, there are clear signs of inflammation, reactive gliosis, and neurodegeneration (Loewen et al., 2016) as well as metabolic and redox dysregulation as reviewed by Pearson-Smith & Patel, 2017. In a zebrafish model of Dravet syndrome, there are metabolic irregularities (Kumar et al., 2016; Banerji et al., 2021) as well as increased astrogliosis associated gene expression (Tiraboschi et al., 2020), which is also apparent in a mouse DS model (Valassina et al., 2022). In pediatric drug-resistant epilepsy, levels of IL-1β in peripheral monocytes correlate to seizure frequency (Yamanaka et al., 2021). Further, an LPS administration model of systemic inflammation in sepsis in conjunction with convulsant PTZ treatment in mice increased seizure susceptibility, which was attenuated with NOX2 genetic ablation and inhibition (Huang et al., 2018). Theiler’s murine encephalomyelitis virus (TMEV) model of temporal lope epilepsy is known to induce neuroinflammation and seizures, but also leads to oxidative stress as indicated by elevated 3-nitrotyrasine levels and reduced GSH/GSSG ratios in the hippocampus (Bhuyan et al., 2015).

Epileptic encephalopathies and genetic epilepsies such as West, Lennox-Gastaut, Dravet, Lafora, and Leigh syndromes have additionally shown evidence of gliosis and neuroinflammation in humans (Kawashima et al., 1999; You et al., 2009; Salar and Galanopoulou, 2018; Lahuerta et al., 2020; Valassina et al., 2022). There is also evidence that most of these types of epilepsies also have a metabolic dysfunction component given the efficacy of the KD in patients (Wijburg et al., 1992; Caraballo et al., 2005, 2014; You et al., 2009). As both neuroinflammation and oxidative stress or metabolic dysregulation are prevalent across the epilepsies, which are both argued to be causal and consequential to the disease, the direct interactions between neuroinflammation and oxidative stress are key components of disease pathology which deserve further evaluation.

## Neuroinflammatory induction of oxidative stress

The role of NADPH oxidases in epilepsy has drawn considerable attention, with findings highlighting a link between oxidative stress production and inflammation. NOX expression of the brain isoforms 1, 2 and 4 are highly inducible, with signs of NOX2 upregulation prevalent in human and animal epilepsies. As described previously, SE can induce ROS production through NOX via NMDA receptor activation. However, NOX expression and activity is also closely linked to neuroinflammation. Pattern recognition receptor (PRR) CR3 and TLR4 signaling by damage associated molecular patterns (DAMPs), such as high mobility group box 1 (HMGB1) released by damaged neurons, is linked to NOX2 expression and activation in microglia (Pei et al., 2007; Gao et al., 2011; Bell et al., 2013; Hou et al., 2018). The inhibition of NF-KB signaling has been shown to reduce LPS induced NOX and inducible nitric oxide synthase (iNOS) expression in various peripheral cell types (Kim B et al., 2008; Al-Harbi et al., 2020; Sul and Ra, 2021). NOX-mediated aberrant ROS production in response to proinflammatory signaling is a major source of oxidative stress in epilepsy, and is further linked to interictal glucose hypometabolism (Malkov et al., 2018). It is suggested that increased NOX activity induced by SE or trauma increases steady-state levels of H_2_O_2_ directly and indirectly by utilizing NADPH needed for glutathione reduction, leading to the inhibition of glycolysis and increased cell susceptibility to oxidative stress. Further, other NOX2-mediated H_2_O_2_ redox signaling is shown to directly increase mtROS production in one of the forms of ROS-induced ROS release (Kim et al., 2017).

DAMPs, particularly HMGB1, have been implicated in epilepsy pathogenesis. HMGB1 and the PRR it can signal through, TLR4, have increased expression in human and animal epilepsies. HMGB1 released from neurons and proinflammatory microglia and has been attributed to both TBI and epilepsy pathogenesis in experimental models as reviewed by Paudel et al., 2018. In animal TLE models, HMGB1 acts through TLR-4 to increase production of cytokines such as IL-1β, TNFα, and IL-6 (Maroso et al., 2010). This TLR-4 mediated signaling cascade can activate NF-KB, leading to the production of these pro-convulsant cytokines. Cytokines like TNFα can, in turn, induce COX-2 and NOX2 gene expression (Newton et al., 1997; Li et al., 2009). Interestingly, peripheral inflammation induced by TLR-4 activation by systemic LPS exacerbated kainic-acid induced seizures and elevated hippocampal reactive microglia, levels of IL-1β, TNFα, and IL-6, and NOX subunit expression (Ho et al., 2015). The ability of PRR-mediated inflammatory signaling to increase NOX expression in epilepsy suggests that enabling ROS production may act as a second messenger to neuroinflammation.

Tumor necrosis factor alpha (TNFα) further impacts the oxidative environment and metabolism in the brain by targeting mitochondria. In neuronal cultures, direct treatment with TNFα resulted in a time dependent decrease in mitochondrial respiration, followed by a reduction in cell viability correlated with increase cytosolic cytochrome c levels (Doll et al., 2015). In mice, systemic LPS increased brain region specific expression of IL-1β as well as mitochondrial complex II/III activity, while decreasing GSH (Noh et al., 2014). Local brain LPS administration, as well as astrocyte exposure to TNFα and IL-1β, depleted GSH (Gavillet et al., 2008; Ariza et al., 2010). These studies indicate that neuroinflammation can influence mitochondrial function, impair antioxidant defenses, and cause oxidative stress to contribute to neurodegeneration.

Neuroinflammation in epilepsy is commonly associated with blood brain barrier (BBB) disruption, allowing infiltration of the brain parenchyma by peripheral immune cells and serum albumin as reviewed in detail by Van Vliet et al. (2015). In addition to exacerbating gliosis and inflammation, BBB disruption also leads to oxidative damage such as in ischemia-reperfusion injury where SOD1 deficiency causes exacerbated neuronal damage and infarct volume (Gasche et al., 2001). Inhibition of matrix metalloproteinase 9 (MMP9), which degrades extracellular matrix around the vasculature, rescues this effect. MMP9 also contributes to parvalbumin positive inhibitory interneuron (PVIN) cell loss that is found in epilepsy. MMP9 is upregulated in epilepsy models, and this ECM proteinase has been shown to degrade the specialized ECM called perineuronal nets (PN) surrounding PVINs (Kim et al., 2009; Acar et al., 2015; Rankin-Gee et al., 2015; Dubey et al., 2017). Interestingly, PVINs are shown to be particularly susceptible to oxidative stress, and the neuroinflammatory based degradation of their neuroprotective PNs leads to oxidative damage and death of these cells (Cabungcal et al., 2013). In fact, neuronal NOX2 expression which is induced by neuroinflammation colocalizes with parvalbumin immunoreactivity in areas of PVIN loss in human TBI (Schiavone et al., 2017).

## Oxidative stress and redox regulation of neuroinflammation

NOX activity may be induced by proinflammatory signaling cascades, but it is also necessary for inducing neuroinflammation. In the kainate and pilocarpine rat experimental models of TLE, ROS production through NOX activity is associated with microglial activation and is responsible for neurodegeneration in these models (Patel et al., 2005; Pestana et al., 2010). SE induced ROS production and neural cell death were attenuated by NOX inhibitors (Pestana et al., 2010; Williams et al., 2015) and overexpression of extracellular SOD (Patel et al., 2005). Interestingly, the deletion of NOX2 following mouse TBI (Dohi et al., 2010; Wang et al., 2018) or PTZ-induced SE (Huang et al., 2018) enhances neurogenesis and reduces cytokine production, indicating that the ROS production occurring following trauma is deleterious to neural recovery. A direct link between NOX activity and inflammation is apparent, as NOX2 mediated production of O_2_^−^ and resulting increased steady-state H_2_O_2_ levels is necessary for NF-KB activation in macrophages (Li et al., 2018) and for microglia proliferation (Mander et al., 2006). The NOX2 inhibitor celastrol attenuated kainate-induced epileptiform activity, indicating that NOX2 is important for seizure initiation (Malkov et al., 2019). As it has been suggested that NOX2 is important for the initial oxidative burst following SE that contributes to seizures in trauma and chemically induced epilepsy models (Zilberter et al., 2022), the efficacy of NOX inhibition in chronic epilepsy and in a clinically relevant window following a seizure inciting event needs further evaluation. Chronic administration of apocynin, a NOX inhibitor antioxidant, following pilocarpine-induced SE did significantly increase neural survival (Lee et al., 2018). Despite the promise of NOX2 inhibition in epilepsy models, further efforts to develop and evaluate clinically translatable BBB permeable compounds such as GSK2795039 are needed (Hirano et al., 2015; Malkov et al., 2019).

Excessive ROS levels are toxic, but at physiological levels ROS such as H_2_O_2_ are important second messengers acting through redox signaling. H_2_O_2_ is an efficient activator of the redox-regulated proinflammatory transcription factor NF-KB, where redox-regulation activates upstream kinases P13K and NIK leading to NF-KB translocation and promotor binding. Further, NOX2 depletion and the NOX inhibitor apocynin were able to reduce NF-KB activation, demonstrating how ROS can lead to the increased expression of proinflammatory cytokines (Kim J et al., 2008). Nrf2, the redox-sensitive transcription factor controlling expression of the antioxidant response element (ARE), is an important modulator of the proinflammatory effects of ROS. For example, nrf2 depletion has been shown to increase NF-ΚB activation, as well as the activation of MMP3/9 and TGF-β following mouse TBI leading to neurodegeneration (Bhowmick et al., 2019). The nrf2 targets heme oxygenase 1 (HO-1), NQO1, and the GSH producing enzymes GCLC and GCLM have all been shown to exert anti-inflammatory effects as reviewed in detail by Ahmed et al., 2017. Not surprisingly, the inhibition of the nrf2 inhibitor KEAP1 with RTA 408, particularly in combination with a NOX inhibitor, prevented cell death and dramatically reduced kainate-induced seizures (Shekh-Ahmad et al., 2019, 2018). The KD’s clinical efficacy in the treatment of various epilepsies may be mediated in part due to its apparent ability to activate nrf2 (Milder et al., 2010). These inflammation modifying roles of nrf2 demonstrate redox-neuroinflammation crosstalk at the transcriptional level which highlights nrf2 as an important therapeutic target for controlling both ROS-mediated damage and activation of proinflammatory cascades.

Certain proinflammatory molecules are also directly regulated by ROS such as IL-1β, HMGB1, and MMP9. IL-1β activation is induced by caspase-1 which is activated by the DAMP-sensitive NLRP3 and NLRC4 inflammasomes (Poyet et al., 2001; Martinon et al., 2002). NLRP3 has been extensively linked to epilepsy, where heightened expression and activation is found in animal and human epilepsies as reviewed by Mohseni-Moghaddam et al., 2021. ROS can lead to NLRP3 activation through binding to thioredoxin-interacting protein (TXNIP) binding following thioredoxin oxidation (Zhou et al., 2011). Further, the crystal structure of NLRP3 revealed a disulfide bond between conserved cysteine residues in the inflammasome active site, suggesting redox-sensitive activity (Bae and Park, 2011). NLRP3 has indeed been shown to be activated by NOX2 mediated ROS production (Abais et al., 2014, 2013). Further, mtROS acts as a second messenger stimulating NLRP3 activation and localization to the mitochondria to promote IL-1β activation, thus inciting a proinflammatory signal in response to mitochondria dysfunction (Zhou et al., 2011; Subramanian et al., 2013). Interestingly, sulforaphane, an nrf2 activator, has been shown to limit mtROS generation and prevent both NLRP3 and NLRC4 activation in murine macrophages (Lee et al., 2016). Although implicated in neurodegenerative disorders but not currently in epilepsy, NLRC4 activation has also been found to be sensitive to mtROS production in astrocytes and contributes to gliosis (Freeman et al., 2017; Samidurai et al., 2020). NLRP3 activation further appears to be inhibited by the activity of the ketone body βHB produced by the KD, suggesting that the modulation of neuroinflammation contributes to KD efficacy (Youm et al., 2015). ROS-mediated inflammasome activation could explain the increased prevalence of IL-1β found in epilepsy as described above, which may in turn perpetuate cycle of oxidative stress and neuroinflammation.

HMGB1 contains three redox sensitive cysteine residues and carries out different immune system roles depending on its redox state. In the reduced state, HMGB1 acts as a chemoattractant. In the oxidized state with two nearby cysteines forming a disulfide bond and the third cysteine unmodified, HMGB1 acts as a proinflammatory cytokine (Yang et al., 2021) which can interact with TLR4 (Balosso et al., 2014). Overoxidation inhibits all HMGB1 functions. Interestingly, combinational drug treatment with sulforaphane and N-actylcysteine (NAC), a compound used to enhance GSH levels, also reduced the production of HMGB1 in the brain and blood in an electrical SE model (Pauletti et al., 2019).

Finally, MMP9 has also been found to have redox-sensitive activation, as oxidants have been shown to directly increase MMP function, contributing to BBB dysfunction (Haorah et al., 2007). IL-1β-induction of MMP9 was dependent on NOX2 activity leading to NF-KB and AP-1 transcription factor activation. Further, this enabled MMP9-dependent astrocyte migration (Yang et al., 2015). This relationship was found to be inhibited by the nrf2 activator RTA 408 (Yang et al., 2019). Incredibly, this redox-sensitive activity of MMP9 is linked to PVIN/PNN irregularities in neurons with impaired GSH synthesis (Gclm^−/−^) *in vivo*. MMP9 inhibition prevented NF-KB activation and restored PVIN/PNN integrity, linking redox-controlled MMP9 to excitatory/inhibitory imbalance (Dwir et al., 2020). Rectifying redox dysregulation in epilepsy may enable the control of these proinflammatory pathways.

In the brain, the major regulator of redox status is GSH, which has also been shown to regulate inflammation. This antioxidant thiol is utilized as the electron donor by glutathione peroxidase (GPx) to convert H_2_O_2_ to H_2_O, so the GSH/GSSG ratio is indicative of the oxidative environment. The expression of enzymes involved in this process such as GCL and GCLM is controlled by nrf2 and NF-KB. Post-translational activation of GCL by dimercaprol in cultured BV-2 microglia prevents LPS-induced production of proinflammatory cytokines and iNOS induction (McElroy et al., 2017a). Depletion of GSH with the GCL inhibitor BSO leads to gliosis and neuroinflammation in the rat brain, with a particular increase in levels of TNFα (González-Fraguela et al., 2018). In this study, this is associated with mild cognitive impairment, suggesting that impaired redox status could contribute to associated comorbidities in epilepsy. GSH in the mitochondria is protective against TNFα-induced neurotoxicity, indicating that GSH depletion in epilepsy may contribute to a vicious cycle of oxidative stress-neuroinflammation-metabolic stress (Fernandez-Checa et al., 1997). Impaired glutathione redox status also enables aberrant oxidative signaling to occur, which can influence redox sensitive ion channels, neurotransmitter receptors, and glutamate reuptake by astrocytes. Restoring GSH redox status may not only control neuroinflammation, but rectify redox based post-translational modifications that can directly influence neural excitability. The therapeutic potential of GSH increasing treatments such as NAC, Coenzyme Q10, sulforaphane, and Vitamin E has begun to be explored in epilepsy models.

Oxidative stress involves the damage to mtDNA, lipids, and proteins in the cell which can lead to neuroinflammation. Astrogliosis is prevalent in the epilepsies, and interestingly a mouse forebrain neuron-specific knockout of SOD2 resulted in seizures, oxidative stress, and marked GFAP and vimentin gene upregulation, indicative of astrogliosis resulting from increased neuronal mtROS (Fulton et al., 2021). Oxidative stress resulting from increased mtROS has significant impacts on redox signaling within neurons (Bao et al., 2009), and can lead to neuronal apoptosis as described in detail by Méndez-Armenta et al., 2014. This oxidative damage originating from neurons can induce neuroinflammation and gliosis through the release of DAMPs. Further, oxidative damage to phospholipids can induce ferroptosis, as described earlier, which has now been directly linked to the induction of neuroinflammation (Cao Y et al., 2021; Cui et al., 2021).

## Discussion

Together, these studies examining oxidative stress, redox dysregulation, metabolic alterations, and neuroinflammation suggest that these processes are all not only involved in the pathogenesis of epilepsy, but fundamentally linked (See Scheme 1). An infection or traumatic brain injury initiates proinflammatory cascades that involve the upregulation and increased activity of NOX. Excessive excitatory signaling induced by status epilepticus or genetic mutations also increase NOX activity. Seizure activity increases metabolic demand in neuronal mitochondria, which increases O_2_^−^ production. The increased oxidative environment resulting from NOX activity and mtROS production leads to GSH depletion. Importantly, NOX utilizes NADPH to produce superoxide, thus depleting NADPH needed by antioxidant systems such as thioredoxin reductase and glutathione peroxidase. Together, these processes exacerbate neuroinflammation through oxidative damage and redox based activation of NF-KB, as well as of HMGB1, inflammasomes and IL-1β, and MMP9. NF-KB activation also increases TNFα, NOX, and COX-2 expression. Neuroinflammatory cytokines such as TNFα cause mitochondrial dysfunction when GSH is depleted, as well as induce reactive astrocytes. This can impair astrocyte regulation of the tripartite synapse, BBB, and neuronal metabolism. Damaged neurons and reactive microglia can increase NOX expression, as well as release MMP9 which degrades ECM around the BBB and PVIN neurons, leaving them susceptible to oxidative damage. Regardless of the initial insult, oxidative bursts through NOX and increased oxidative phosphorylation in the mitochondria induce metabolic alterations responsible for observed interictal glucose hypometabolism. Glycolysis further fuels the pentose phosphate pathway (PPP) which produces NADPH. Together, oxidative stress and redox dysregulation form a vicious cycle with neuroinflammation that likely underlies epileptogenesis and undermines current treatment strategies.

Developing combinational therapies that address neuronal hyperexcitability as well as oxidative stress may prevent the deleterious cycle of neuroinflammation and oxidative stress that contribute to epileptogenesis. Recent advances have identified redox-based treatment strategies that warrant further investigation (Yang et al., 2020; Parsons et al., 2022). NOX-inhibitor therapy has shown neuroprotective effects in numerous epilepsy models. However, research suggesting that NOX2 is primarily responsible for the oxidative burst immediately following SE may not allow for a wide enough treatment window in patients following their first SE event. Inhibited interictal glycolysis may contribute to a more oxidative environment due to the decreased substrate pool for the PPP. Interestingly, the KD pushes metabolism away from glycolysis, potentially removing fuel needed to trigger seizures while introducing ketone bodies. This can diminish neuroinflammation and induce Nrf2 activation to express the ARE, which has proven efficacy in treating epilepsy patients. Indeed, Nrf2 activators such as sulforaphane and hydroxylated fullerene have restored GSH levels and shown antiepileptic and neuroprotective properties in numerous epilepsy models (Wang et al., 2014; Carrasco-Pozo et al., 2015; Pauletti et al., 2019; Uddin et al., 2020; Cao H et al., 2021; Sandouka and Shekh-Ahmad, 2021). An antioxidant and metal chelator, curcumin, was able to reduce gliosis and cytokine levels in PTZ-induced epilepsy (Kaur et al., 2015). Other redox-based therapies such as AEOL10150, salicylamine, Coenzyme Q10, and NAC have also been shown to restore GSH homeostasis, which is critical to control inflammation, leading to reduction of seizure burden or comorbidities in animal models (Shin et al., 2005; Tawfik, 2011; Pearson et al., 2015; McElroy et al., 2017b). Further, in a small study of patients with refractory epilepsy, Vitamin E in combination with ASMs increased total antioxidant capacity, catalase, and glutathione and reduced seizure frequency (Mehvari et al., 2016). It is herein proposed that tempering excessive ROS production and reducing oxidative stress could thereby control neuroinflammatory processes involved in epilepsy. A comprehensive treatment strategy broadly effective in epilepsy may therefore involve a combinational approach of ASMs coupled with GSH inducing and nrf2 activating compounds.
